# Ketogenic diet aggravates cardiac remodeling in adult spontaneously hypertensive rats

**DOI:** 10.1186/s12986-020-00510-7

**Published:** 2020-10-26

**Authors:** Yuehua You, Yongzheng Guo, Ping Jia, Biaobiao Zhuang, Yu Cheng, Hongpei Deng, Xiaowen Wang, Cheng Zhang, Suxin Luo, Bi Huang

**Affiliations:** 1 Division of Cardiology, The First Affiliated Hospital of Chongqing Medical University, Chongqing, 400016 China; 2 Institute of Life Science, Chongqing Medical University, Chongqing, 400016 China; 3 Department of Cardiothoracic Surgery, The First Affiliated Hospital of Chongqing Medical University, Chongqing, 400016 China

**Keywords:** Ketogenic diet, Cardiac fibrosis, Hypertension, mTOR pathway, mTORC2

## Abstract

**Background:**

Ketogenic diet (KD) has been proposed to be an effective lifestyle intervention in metabolic syndrome. However, the effects of KD on cardiac remodeling have not been investigated. Our aim was to investigate the effects and the underling mechanisms of KD on cardiac remodeling in spontaneously hypertensive rats (SHRs).

**Methods:**

10-week-old spontaneously hypertensive rats were subjected to normal diet or ketogenic diet for 4 weeks. Then, their blood pressure and cardiac remodeling were assessed. Cardiac fibroblasts were isolated from 1- to 3-day-old neonatal pups. The cells were then cultured with ketone body with or without TGF-β to investigate the mechanism in vitro*.*

**Results:**

4 weeks of KD feeding aggravated interstitial fibrosis and cardiac remodeling in SHRs. More interestingly, ketogenic diet feeding increased the activity of mammalian target of rapamyoin (mTOR) complex 2 pathway in the heart of SHRs. In addition, β-hydroxybutyrate strengthened the progression of TGF-β-induced fibrosis in isolated cardiac fibroblasts. mTOR inhibition reversed this effect, indicating that ketone body contributes to cardiac fibroblasts via mTOR pathway.

**Conclusions:**

These data suggest that ketogenic diet may lead to adverse effects on the remodeling in the hypertensive heart, and they underscore the necessity to fully evaluate its reliability before clinical use.

## Introduction

Hypertension is one of the most prevalent chronic diseases and has been the most important risk factor for cardiovascular diseases worldwide [1], including stroke, heart failure, and aneurysm, etc. [2]. Therefore, finding ways to keep blood pressure under control is vital for reducing the risk of cardiovascular events. As hypertension is one of the most important characteristics of metabolic syndrome, lifestyle interventions such as exercise, weight loss and reasonable dietary patterns are a standard and first-line way to manage and treat hypertension [3–5]. Ketogenic diet (KD), which is very low in carbohydrates and high in fats or protein, has been used as an approach to lose body weight and improve metabolic syndrome [6, 7]. However, besides the benefits, KD has been reported to induce some adverse effects such as hepatic steatosis and dyslipidemia [8]. Our previous study also showed that KD leads to dyslipidemia [9, 10], which is a risk factor for the development of hypertension. However, studies concerning on the potential effects of KD on cardiac remodeling are scare.

Cardiac remodeling, characterized by interstitial fibrosis and cardiomyocyte hypertrophy, is one of the most serious complications contributing to failing heart in hypertension [11]. Many pathological processes and signal pathways are involved in the development of cardiac remodeling in hypertension [12, 13]. Over the past years, strong evidence has suggested that the mammalian target of rapamyoin (mTOR) overactivation plays important roles in the fibrosis process, and suppressing mTOR is a target for preventing hypertension and its related fibrosis [14]. mTOR is a serine/threonine kinase belonging to PI3K ([phosphoinositide 3-kinase]-related kinase family), which could integrate energy, nutrient and growth factor signals to regulate cell proliferation, cell growth and inflammatory responses [15]. There are two different protein complexes of mTOR, namely complex1 (mTORC1) and complex 2 (mTORC2) [16] . The phosphorylation of their final downstream effector ribosomal protein S6 is the functional marker of mTORC1 kinase activity and the phosphorylation of Protein Kinase B (Akt) is the functional marker of mTORC2 [17]. Our previous study reported that ketogenic diet could activate the phosphorylation of Akt in diabetic mice, which indicates that KD may contribute to the activation of mTOR pathway and exert adverse effects on cardiac remodeling [9]. Therefore, in the present study, we investigated the effects of KD on cardiac remodeling and mTOR pathway in hypertensive rats.

## Methods

### Animals

Spontaneously Hypertensive Rats (SHRs) and Wistar rats (WT) were obtained from Beijing Vital River Laboratory Animal Technology Company. Male rats were used for both in vivo and vitro experiments. All rats were maintained on a 12-hour light/dark schedule. Rats were randomly divided into two groups receiving normal diet (ND) or KD for 4 weeks, starting at the age of 10 weeks. All study protocols were approved by The Institutional Animal Care and Use Committees of Chongqing Medical University.

### Rats’ diets and feeding

Food and water were provided ad libitum. The percentage of calories from macronutrient content is as follows: ND, 10 % from protein, 13 % from fat and 77 % from carbohydrates; KD, 10 % from protein and 90% from fat. All diets are matched on a per-calorie basis for micronutrient content, fiber and preservatives. The ingredients of experimental diets are shown in Additional file 1: Table S1.

### Blood pressure

The blood pressure and heart rate were measured with a noninvasive computerized tail-cuff blood pressure system as previously described [18]. Briefly, the rats were allowed to calm down in a tunnel maintaining at 37 ℃, then the blood pressure and heart rate were collected for 3 times. The mean values were taken as the final blood pressure and heart rate of rats.

### Blood sampling

WT and SHR were anesthetized with 3% isoflurane in 100% oxygen. Blood samples were collected from the abdominal aorta with a sterile tube and centrifuged at 3000 RPM for 10 minutes at room temperature. Then the serum was collected and stored at −80 ℃.

### Determination of heart reduced glutathione (GSH), malondialdehyde (MDA), manganese superoxide dismutase activity (MnSOD) activity

The cardiac GSH, MDA levels and SOD activities were measured with commercial assay kits (Nanjing Jiancheng Reagents, Jiangsu, China) according to the manufacturer’s instructions.

### Measurement of intramyocardial β-hydroxybutyrate (β-OHB)

The left ventricular tissues were homogenized and the intramyocardial β-OHB content was measured with β-hydroxybutyrate Assay Kits (Nanjing Jiancheng Reagents, Jiangsu, China) according to the manufacturer’s instructions.

### Histology and staining

Hearts were fixed with 4% paraformaldehyde, embedded in paraffin, and 7-μm-thick sections were used for staining. Masson trichrome staining was used to measure interstitial fibrosis of the hearts with commercial kits (Solarbio, Beijing, China) according to the manufacturer’s instructions. WGA (Invitrogen, USA) staining was used for the analysis of the cardiomyocyte area. The images of Masson and WGA were taken from left ventricular. Eosinophilic infiltration was measured by myeloperoxidase (MPO) staining. For the measurement of mitochondrial ROS, 5 μM mitoSOX (Invitrogen, USA) was loaded for 15 minutes and washed. Immunohistochemical staining of CD68 was performed to assess macrophage accumulation. Sections were incubated with primary antibody (anti-CD68, 1:100, ab125212, Abcam, USA) overnight at 4°C, and then incubated with corresponding secondary antibody for 40 min at 37 °C. After then, the sections were immersed in 3,3′diaminobenzidine (DAB) at room temperature for 30s. The pictures were collected randomly with a Zeiss DM4B microscope.

### Cardiac fibroblasts culture

Neonatal cardiac fibroblasts were isolated from 1- to 3-day-old neonatal pups as previously described [19]. Briefly, hearts from neonatal rats were harvested and cut into 1–3 mm^3^ pieces. Then, they were washed 3 times with PBS, followed by digestion with trypsin and collagenase at a 15 ml centrifugal tube. The digestion was terminated with DMEM medium containing 10 % FBS. After centrifugation at 1000 RPM for 5 minutes at 20–25 ℃, cells were transferred into a culture flask, then the medium was abandoned and the adhered cells were considered as cardiac fibroblasts. 10 µM Y27632, a Rho-associated kinase (ROCK) inhibitor (MCE,USA), was used to prevent the transformation to activated fibroblast. The medium with Y27632 was replaced when cardiac fibroblasts were exposure to TGF-β and β-OHB. All fibroblasts were used between passage 2 and 3.

### Cell proliferation assay

Cell proliferation was measured using Cell Counting Kit-8 (Biosharp, Shanghai, China) as previously described [20]. Briefly, cells were cultured in a 96-well plate, and 10μl of CCK-8 solution was added to the medium and incubated for 2h. The absorbance at 450nm wave length was determined with a reference wave length of 570nm.

### Western blots

The heart tissues or cardiac fibroblasts were lysed with RIPA with protease inhibitor and phosphatase inhibitor cocktail. Samples were incubated with blue loading buffer for 10 minutes at 100 ℃. 20–40 μg samples were separated by SDS/PAGE gels, then transferred to a PVDF membrane. After being blocked with 5 % non-fat milk in TBS with 0.1 % Tween 20, the membranes were incubated with the primary antibodies overnight at 4 ℃ followed by secondary antibodies for 1 hour at room temperature. The following antibodies were used for immunoblotting: Collagen1 (14695), Collagen3 (22734), SOD2 (24127), NOX4 (14347), MCP-1(66272) (Proteintech), p-Akt^473^(4060), Akt(9272S), p-AMPK(4184), AMPK(5832) (Cell Signaling Technology), p-S6^ser235^ (AF3354), S6 (AF6354) and GAPDH (AF7021) (Affinity). Images were visualized using ECL Western Blotting System (Bio-Rad, CA, USA).

### Quantitative real-time PCR

Total RNA was isolated from using RNAiso plus (Takara, Japan) and reverse transcribed to complementary DNA (cDNA) using the PrimeScript RT reagent kit (Takara, Japan). Then the total cDNA was amplified and analyzed by SYBR Green PCR Master Mix (Takara, Japan) in a CFX96 Real-time System (Bio-Rad, USA). The sequences of rat-specific primers for tumor necrosis factor-α (TNFα), IL-1β, interferon γ (IFN-γ), and GAPDH used in the study are listed in Table S2.

### Statistical analysis

All data were expressed as mean ± SEM. Statistical analyses were performed by two-way ANOVA with Tukey multiple-comparison tests. P values less than 0.05 were considered to be significant. All statistical analyses were performed with GraphPad Prism 7 software.

## Results

### Ketogenic diet contributes to hypertension in SHRs

**Fig. 1:**
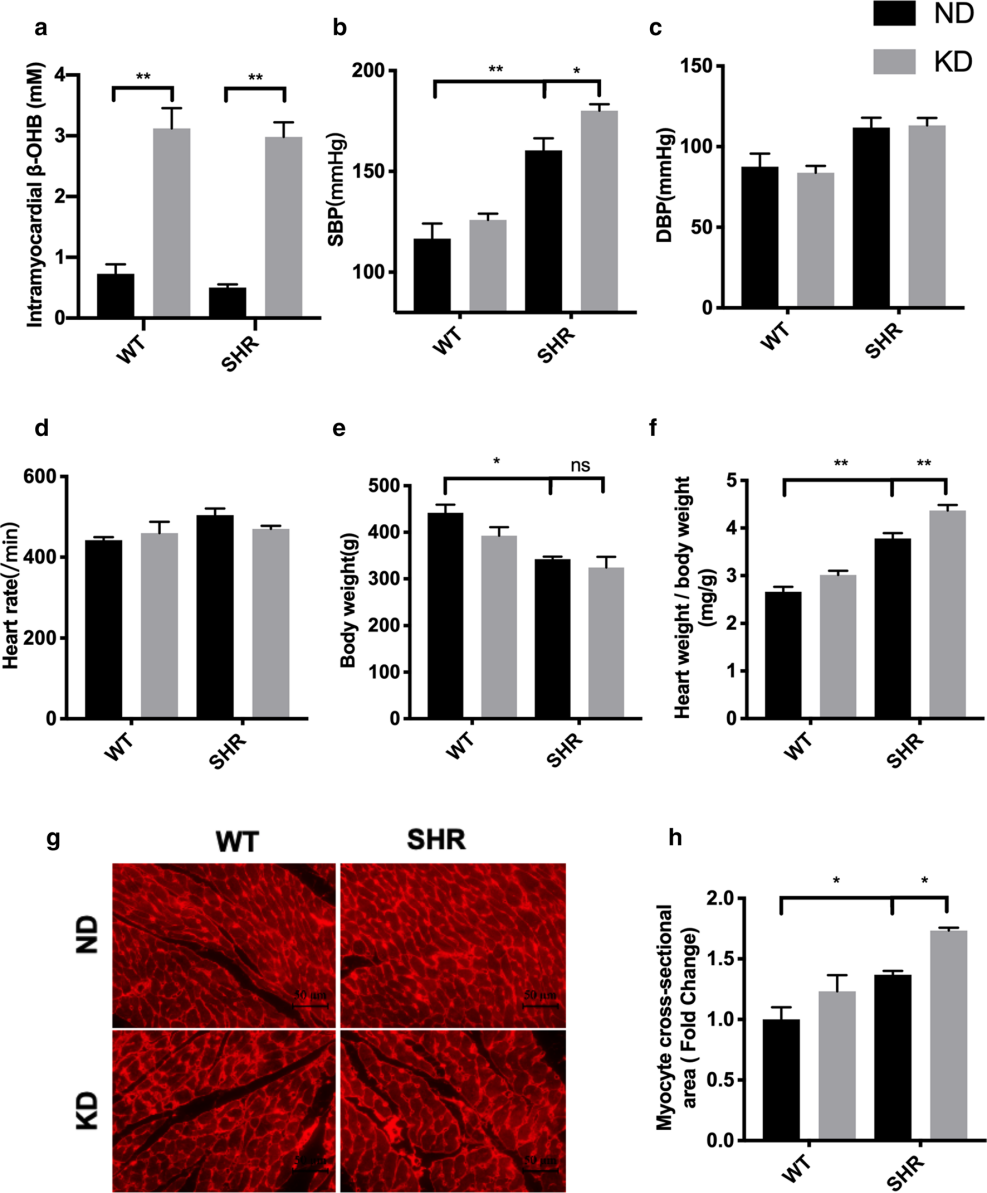
Ketogenic diet contributed to hypertension and cardiac remodeling in spontaneously hypertensive rats. **a** Ketogenic diet (KD) increased the intramyocardial β-hydroxybutyrate content. **b** systolic blood pressure (SBP), diastolic blood pressure (DBP). c heart rate. **d** and body weight. **e** were measured. **f** KD feeding increased ration of heart weight-to-body weight of spontaneously hypertensive rats. **g** Cardiomyocyte cross-sectional area in left ventricular tissue was measured by WGA. Scale bar, 50 μm. The statistical results were shown in H. Values are the mean ± SEM. n = 4 for each ND group. n = 8 for each KD group. *P < 0.05, **P < 0.01

To investigate the effects of ketogenic diet on blood pressure and cardiac remodeling, rats were subjected to a ND or KD for 4 weeks, starting at the age of 10 weeks. The composition of KD is showed in Additional file 1: Table S1. As expected, KD feeding significantly increased the β- hydroxybutyrate contents in hearts, suggesting that ketosis was reached in hearts by KD (Fig. 1a). Compared with age-matched Wistar rats, SHRs showed an increased systolic blood pressure (SBP), which was further enhanced by KD feeding (Fig. 1b). However, diastolic blood pressure (DBP) and heart rate were not markedly affected by KD treatment (Fig. 1c, d). Although KD feeding reduced the body weight of Wistar rats, the body weight between SHRs groups were comparable (Fig. 1e). In addition, KD feeding increased cardiac hypertrophy as evidenced by increased ration of heart weight-to-body weight (Fig. 1f) and myocyte cross-sectional area (Fig. 1g–h) in SHRs. Taken together, these data suggest that ketogenic diet aggravates hypertension and cardiac remodeling in SHRs.

### Ketogenic diet aggravates interstitial fibrosis in the heart of SHRs

**Fig. 2:**
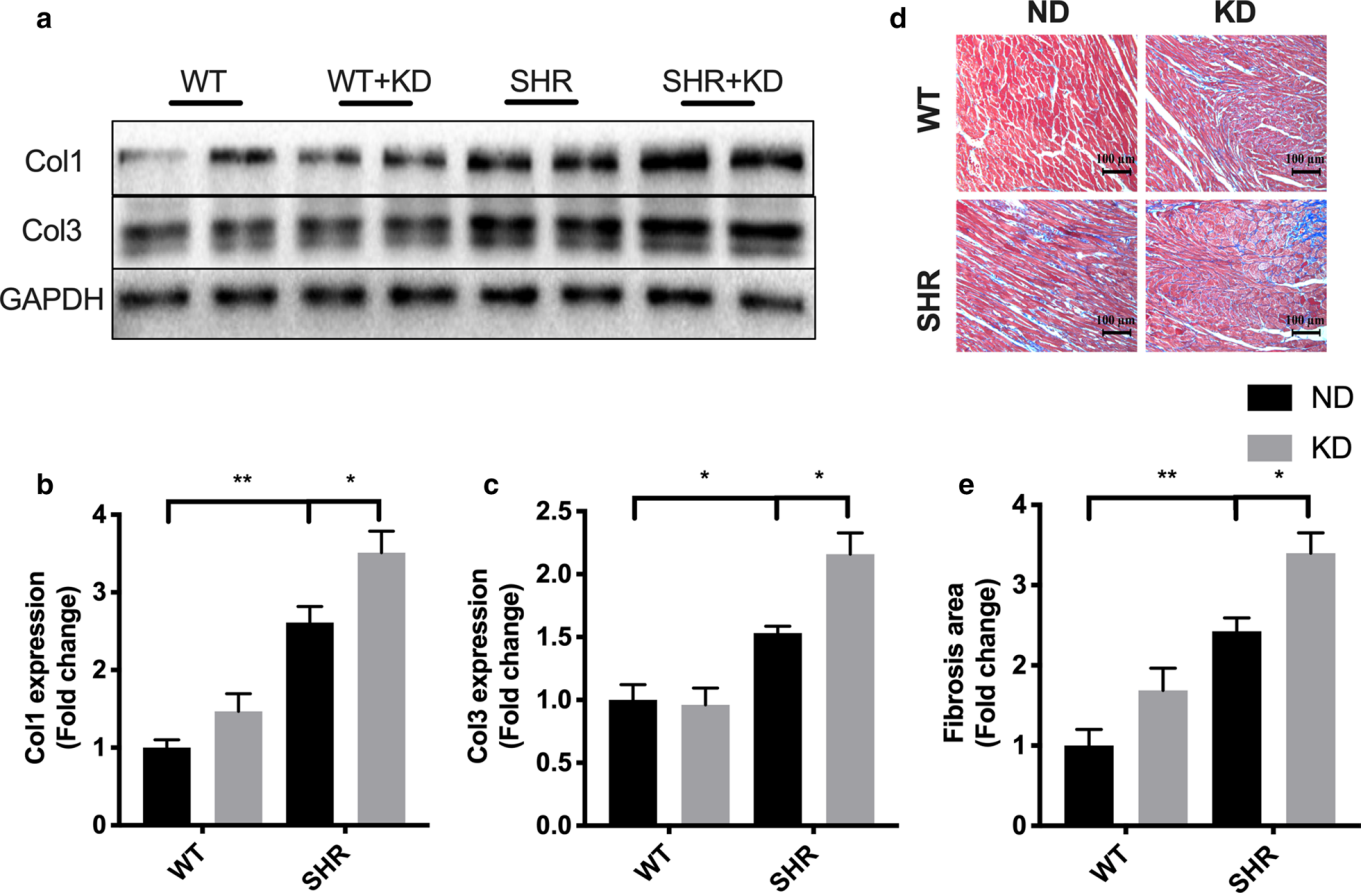
Ketogenic diet aggravated interstitial fibrosis in the heart of spontaneously hypertensive rats. **a** Ketogenic diet increased the expression of collagen in the heart of spontaneously hypertensive rats. The statistical results are shown in **b** and **c**. **d** Masson trichrome stain was used on the left ventricular tissue to visualize interstitial fibrosis in hearts. Scale bar, 100 μm. The statistical results are shown in E. Values are the mean ± SEM. n = 4 for each group. *P < 0.05, **P < 0.01

As interstitial fibrosis is an important factor responsible for cardiac remodeling, we next performed experiments to determine whether KD feeding contributed to interstitial fibrosis in the hypertensive hearts. Compared with WT rats, SHRs exhibited increased cardiac interstitial fibrosis as evidenced by elevated collagen expression. Moreover, the collagen expression was further increased by feeding KD (Fig. 2a–c). We also performed Masson’s trichrome stain to evaluate the interstitial fibrosis in heart. The results showed that the blue staining which represents fibrosis were significantly increased in hypertensive hearts by KD feeding (Figure D and E). These findings indicate that interstitial fibrosis is responsible for the aggravated cardiac remodeling in SHRs subjected to KD feeding.

### Ketogenic diet promotes oxidative stress injury in SHRs

**Fig. 3:**
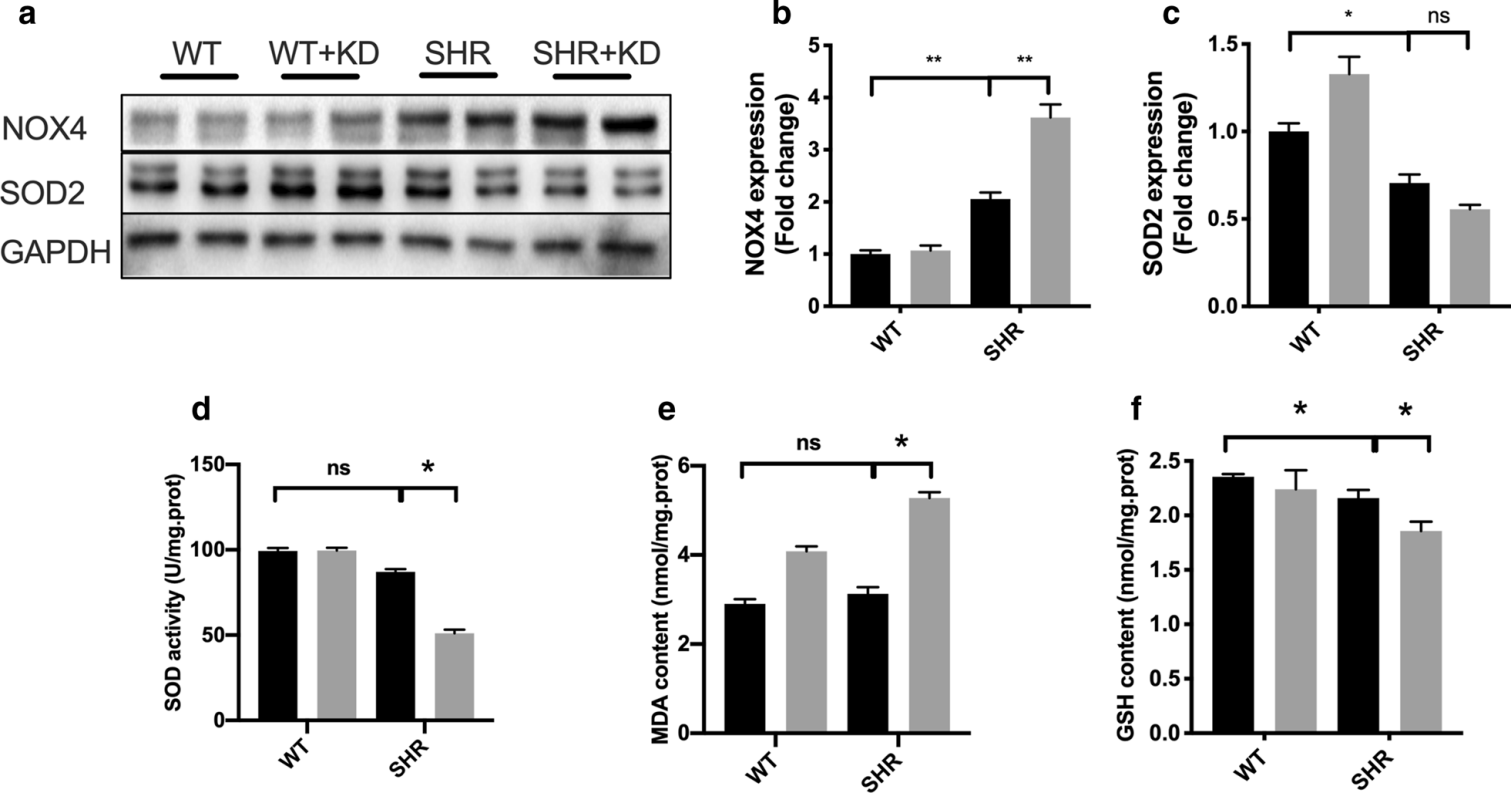
Ketogenic diet promoted oxidative stress injury in spontaneously hypertensive rats. **a** The expression of SOD2 and NOX4 in the heart was measured by western blotting. The quantified SOD2 and NOX4 results are shown in **b** and **c**. **d** ketogenic diet decreased the SOD activity and increased the malondialdehyde (MDA) contents **e** in the heart of SHRs. **f** ketogenic diet decreased the GSH contents in the heart of SHRs. Values are the mean ± SEM. n = 4 for each group. *P < 0.05, **P < 0.01

It has been reported that oxidative stress plays a vital role in cardiac remodeling and the activation of cardiac fibroblasts [21]. Our results showed that ketogenic diet significantly increased mitochondrial ROS production in the heart of SHRs (Fig. 3a, b). We thus measured the expression of NADPH Oxidase 4 (NOX4), which catalyzes the reduction of molecular oxygen to reactive oxygen species, and the expression and activity of antioxidant enzyme SOD2. As expected, KD treatment significantly increased the expression of NOX4 (Fig. 3a, b) in SHRs. Although KD didn’t affect the expression of SOD2 significantly in hearts (Fig. 3c), the activity of SOD2 (Fig. 3e) in heart were markedly suppressed by feeding KD. Furthermore, KD feeding reduced the glutathione (GSH) content (Fig. 3f) however increased the malondialdehyde (MDA) content in heart of SHRs (Fig. 3g), also proving that KD promoted oxidative stress injury in SHRs.

### Ketogenic diet enhances inflammatory responses in the heart of SHRs

**Fig. 4:**
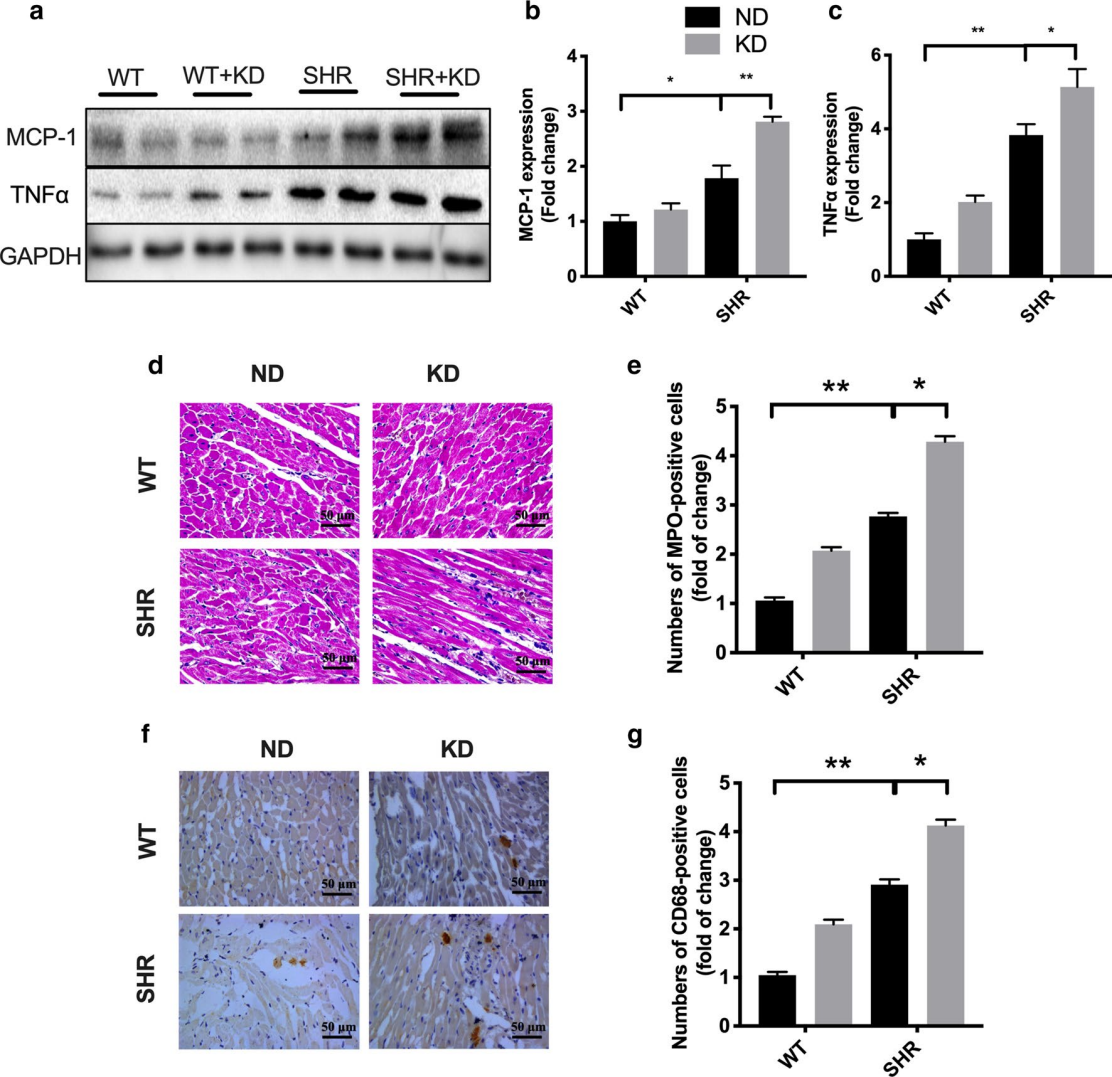
Ketogenic diet enhanced inflammatory responses in the heart of spontaneously hypertensive rats. **a** Ketogenic diet increased the expression of MCP-1 and TNFα in the heart of SHRs. The quantified MCP-1 and TNFα results are shown in **b** and **c**. **d**–**e** Heart sections were stained with myeloperoxidase to evaluate eosinophilic infiltration. **f**–**g** Heart sections were Immunohistochemical staining of CD68 to evaluate macrophage accumulation. Values are the mean ± SEM. n = 4 for each group. *P < 0.05, **P < 0.01

It is widely accepted that imynflammatory responses play a central role in provoking interstitial fibrosis, and ketogenic diet could exert various effects on inflammation in different tissues [22]. Therefore, we evaluated the effects of KD on the expression of inflammatory markers in the heart of SHRs. Monocyte chemoattractant protein 1 (MCP-1) contributes to the infiltration of monocytes, memory T cells and dendritic cells into injury tissues and produces inflammatory factors. Compared with WT rats, the expression of MCP-1 and TNFα was significantly increased in the heart of SHRs and KD feeding further promoted the expression of MCP-1 and TNFα (Fig. 4a–c). KD also increased neutrophil (MPO+) (Fig. 4d–e) and macrophage (CD68+) (Fig. 4f, g) infiltration in hearts of hypertensive rats as well as the transcription of IL-1β, TNFα and IFN-γ in hearts (Additional file 1: Figure S1). All of those evidences indicate that ketogenic diet aggravates the inflammatory responses in the heart of SHRs.

### Ketogenic diet activates mTORC2 in the heart of SHRs

**Fig. 5:**
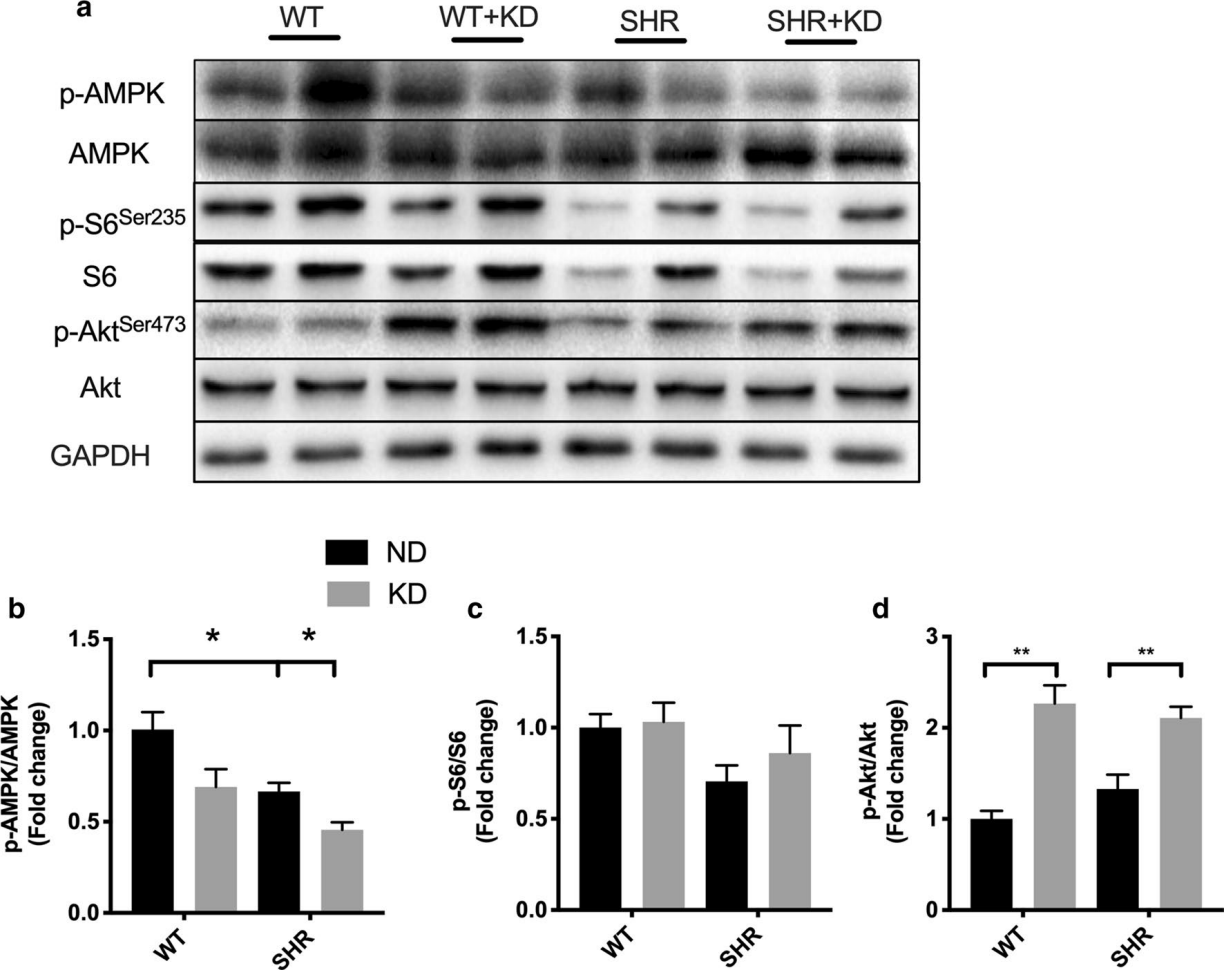
Ketogenic diet activated mTORC2 in the heart of spontaneously hypertensive rats. **a** The phosphorylation of AMPK and the ratio of p-S6/S6 and p-Akt/Akt in the heart of Wistar rats and SHRs were measured with Western blots. The quantitative result were shown in **b**–**d**. Values are the mean ± SEM. n = 4 for each group. *P < 0.05, **P < 0.01

It has been demonstrated that AMPK-mTOR pathway, which is affected by energy and nutrition supply, plays a major part in activating interstitial fibrosis and inflammatory response [23]. KD treatment significantly suppressed the phosphorylation of AMPK evidenced by Western blots (Fig. 5a, b). We next measured the activity of mTOR. The ratio of phospho (p)S6^S235^/S6 is the functional marker of mTORC1 kinase activity and the ratio of p-Akt^S473^/Akt is the functional marker of mTORC2 kinase activity. We can conclude that both mTORC1 and mTORC2 activity were comparable between normal and hypertensive hearts, and that KD treatment didn’t affect the activity of mTORC1, however increased the activity of mTORC2 in the heart of both WT and SHRs (Fig. 5c, d). These results suggest that ketogenic diet activates mTORC2 and this may be responsible for increased fibrosis and inflammation in hearts of SHRs.

### β-hydroxybutyrate strengthens the progression of TGF-β-induced fibrosis via mTOR pathway in isolated cardiac fibroblasts

**Fig. 6:**
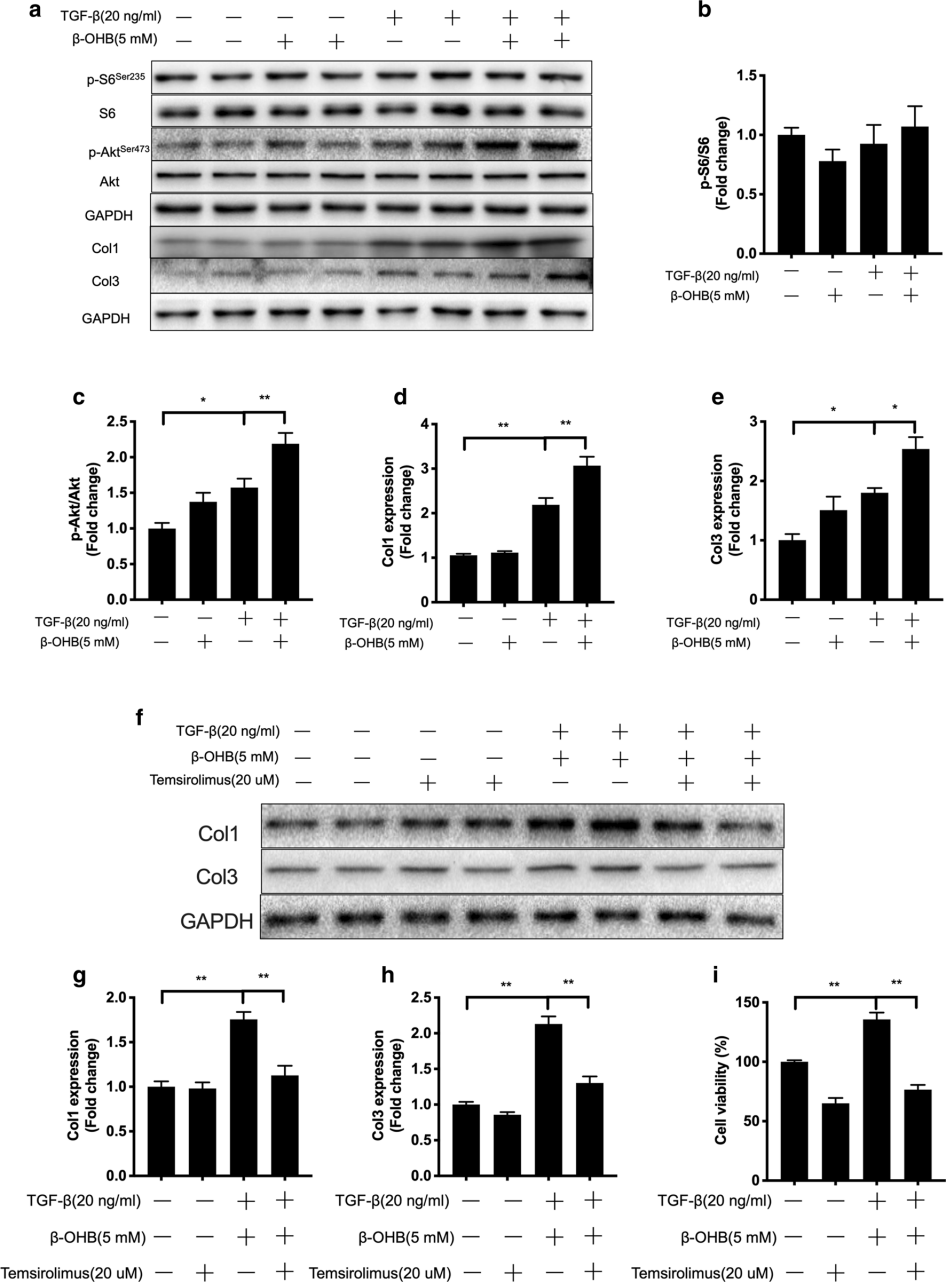
β-hydroxybutyrate strengthened the progression of TGF-β-induced fibrosis via mTOR pathway in isolated cardiac fibroblasts. **a** Cardiac fibroblasts were incubated with β-hydroxybutyrate (β-OHB) and TGF-β. Then the activation of mTOR and the expression of collagen were measured. The quantified results are shown in **b**–**e**. **f**–**h** The effects of co-stimuli of β-OHB and TGF-β in cardiac fibroblasts were blunted by the mTOR inhibitor Temsirolimus. **i** inhibition of mTOR with Temsirolimus suppressed β-OHB and TGF-β-induced proliferation in cardiac fibroblasts. Values are the mean ± SEM. n = 4–6 for each group. *P < 0.05, **P < 0.01

β-hydroxybutyrate (β-OHB) is an endogenic metabolite that is synthesized in the liver from fat, and it circulates throughout the body for energy supply [24]. Our data showed KD enhanced the ketone body β-OHB content in hearts (Fig. 1a). To further verify our hypothesis, we further investigated whether β-OHB could activate mTORC2 in cardiac fibroblasts. It was found that the ketone body β-OHB treatment for 24 hours didn’t induce the activation of mTORC1 or mTORC2 (Fig. 6a–c). And the expression of collagen was not affected by KD feeding, suggesting that β-OHB alone can’t activate mTOR or contribute to the fibrogenesis in cardiac fibroblasts. Transforming growth factor β (TGF-β) is a central mediator of cardiac fibrosis and plays a vital role in hypertensive cardiac remodeling [11]. To mimic the in vivo environment, we exposed fibroblasts to β-hydroxybutyrate and TGF-β co-stimulation. It was shown that β-OHB and TGF-β co-treatment didn’t affect the ratio of p-S6/S6 (Fig. 6b), but significantly increased the ratio of p-Akt/Akt (Fig. 6c), suggesting an increased activity of mTORC2. Similarly, the expression of collagen 1 and collagen 3 were also increased in cardiac fibroblasts exposed to co-stimulation by β-hydroxybutyrate and TGF-β (Fig. 6d, e). More importantly, these effects were almost eliminated by mTOR inhibition with Temsirolimus (Figure F–H). In addition, proliferation is another feature of activated cardiac fibroblasts. β-OHB alone didn’t induce the proliferation of cardiac fibroblasts. TGF-β could increases cell viability but this effect couldn’t be blunted by mTORC inhibitor (Fig. 6I). Compared with only TGF-β, β-hydroxybutyrate and TGF-β co-stimulation further activated proliferation, and inhibition of mTOR suppressed β-hydroxybutyrate and TGF-β-induced proliferation in cardiac fibroblasts (Fig. 6I). Taken together, these results indicates that β-OHB strengthenes the fibrosis progression induced by TGF-β via mTOR pathway in cardiac fibroblasts.

## Discussion

Historically, dietary strategies that restrict carbohydrate have been used for weight loss. Multiple studies have outlined the successful use of the ketogenic diet in obesity through an increase in satiety. Ketone bodies are the major source of energy in the periods of fasting and/or carbohydrate shortage and play a role in appetite suppressant through actions on specific hypothalamic neurons and increasing the release of leptin [25, 26]. In addition, reduced carbohydrate intake results in decreased insulin levels and blood glucose concentrations, which has been hypothesized to produce metabolic benefits [27, 28]. However, there are contradictory data about KD. Despite their beneficial effects on weight loss, metabolic diseases and epileptic seizures, it has been recently demonstrated that a long-term KD treatment caused hepatic steatosis, a pro-inflammatory state, pancreatitis and in patients [29]. Our previous study also showed that ketogenic diet might lead to dyslipidemia [9], which is a strong risk factor for cardiovascular diseases. Hypertension is a primary risk factor for cardiovascular disease and one of the main characteristics of metabolic syndrome. However, the potential role of ketogenic diet in hypertension has received scarce attention.

Here, it was shown that 4 weeks of KD feeding contributed to interstitial fibrosis and aggravated cardiac remodeling in SHRs. In addition, as an endogenic metabolite of KD, β-OHB strengthened the progression of TGF-β-induced fibrosis in cardiac fibroblasts. Inhibition of mTOR pathway almost reversed these effects, suggesting that ketone body might contribute to cardiac fibroblasts via mTOR pathway in hypertension. These data suggested that ketogenic diet may lead to negative effects on hypertension, which underscores the necessity to fully evaluate its reliability before using it for people who are suffering from hypertension.

The pathogenesis of hypertension-related cardiac remodeling is very complex and its underlying mechanisms remain to be further elucidated. However, oxidative stress and inflammatory responses have been confirmed to be involved in the progress of cardiac remodeling [30–32]. Accordingly, our previous study also suggested that mitochondria dysfunction and increased oxidative damage were associated with cardiac remodeling in TAC mice [33]. Here, we examined the effects of KD on oxidative stress and inflammation in SHRs. Our data showed that inflammation and oxidative stress were increased in hypertensive heart, and KD further enhanced these adverse effects, which may be responsible for the aggravated cardiac remodeling in SHRs receiving KD feeding.

AMPK-mTOR pathway has been noticed by people in the physiological process of hypertension and cardiac remodeling [34, 35]. AMPK, as the upstream molecules of mTOR, can inhibit the activity of mTOR. Actually, the effect of the ketogenic diet on AMPK-mTOR activity is of tissue specific effects and affected by leptin and insulin [36]. AMPK activity increased in the liver and adipose tissue but decreased in the hypothalamus and heart. In the present study, we also observed a decreased AMPK activity in KD-treated SHRs, which is in line with previous study [37]. It has been found that mTOR play roles both in oxidative stress and inflammatory responses [14]. In our present study, we found that KD increased the activity of mTORC2 but didn’t affect the activity of mTORC1, which indicated that KD may contribute to oxidative and inflammation via activating mTORC2. In accordance with this, previous studies reported that increased levels of PI3K/Akt/mTOR were found in the kidney of a rat model with DOCA-salt-induced hypertension, and inhibition of mTORC2 completely prevented salt-induced hypertension in rats [17, 38]. These studies, combined with ours, all indicated that mTORC2 may play an important role in cardiac remodeling and hypertension.

Excessive cardiac fibrosis is a major reason for cardiac remodeling. The activation and excessive proliferation of cardiac fibroblast is the major reason for cardiac fibrosis [39]. It is important to figure out whether KD-mediated mTOR activation can contribute to the pro-fibrotic effects of cardiac fibroblast. Interestingly, we found β-OHB alone couldn’t activate the mTORC2, thereby unable to induce increased expression of collagen in cardiac fibroblasts. Given that transforming growth factor β (TGF-β) is the main initiator of fibrosis in hypertensive situation in vivo, we next exposed cardiac fibroblasts to β-OHB and TGF-β co-stimuli. The results showed that β-OHB enhanced TGF-β-induced activation of mTORC2 and expression of collagen in cardiac fibroblasts. In addition, inhibition of mTOR completely prevented the increased expression of collagen in cardiac fibroblasts. These data suggest that ketogenic diet may only contribute to pro-fibrotic actions via mTOR pathway in hypertensive situation, which is in line with the result of our previous study that KD didn’t induce fibrosis in diabetic mice (Fig. 2). And another study also reported that KD strengthen the fibrogenic response in hepatic stellate cells via TGF-β [40], but the mechanisms underlying how β-OHB stimulates TGF-β still deserves further study.

There are some limitations needed to be pointed out in this study. First, because no selective inhibitor is available yet, we chose Temsirolimus, which inhibits both mTORC1 and mTORC2, for our experiments in vitro. Thus, we can’t exclude the effects of mTORC1 on the activation of cardiac fibroblasts. Second, we only investigated the pro-fibrotic mechanisms of ketogenic diet in vitro, but the effects in vivo still deserve further study.

## Conclusions

Taken together, the present study suggests that ketogenic diet aggravates cardiac remodeling in SHRs via activating mTOR pathway. Therefore, its reliability should be given full evaluation before using it for hypertensive individuals.

## Supplementary information

**Additional file 1: Table S1:** Ingredients of experimental diets. **Table S2:** Primers for tumor necrosis factor-α (TNFα), IL-1β, interferon γ (IFN-γ), and GAPDH. **Figure S1:** qRT-PCR was performed to measure the expression of IL-1β, TNF-α, and IFN-γ in the heart of WT and SHRs.

## Data Availability

All data, analytic methods, and study materials presented within this article and in the Data Supplement are available for other investigators from the corresponding authors on reasonable request.

## Abbreviations

KD: Ketogenic diet
ND: Normal diet
SHRs: Spontaneously hypertensive rats
WT: Wistar rats
β-OHB: β-Hydroxybutyrate
mTOR: Mammalian target of rapamycin
mTORC1: MTOR complex1
mTORC2: MTOR complex2
PI3K: Phosphatidylinositol 3 kinase
Akt: Protein kinase B
MDA: Malondialdehyde
MnSOD: Manganese superoxide dismutase activity
DBP: Diastolic blood pressure
SBP: Systolic blood pressure
NOX4: NADPH oxidase 4
MCP-1: Monocyte chemoattractant protein 1
TNFα: Tumor necrosis factor α
TGF-β: Transforming growth factor β
