# Supplementary material for: Ketogenic diet aggravates cardiac remodeling in adult spontaneously hypertensive rats
Source: Nutr Metab (Lond). 2020 Oct 26;17:91. doi: 10.1186/s12986-020-00510-7 (PMC7586698; doi:10.1186/s12986-020-00510-7)
Supplement: Supplementary file 1 — Additional file 1: Table S1: Ingredients of experimental diets. Table S2: Primers for tumor necrosis factor-α (TNFα), IL-1β, interferon γ (IFN-γ), and GAPDH. Figure S1: qRT-PCR was performed to measure the expression of IL-1β, TNF-α, and IFN-γ in the heart of WT and SHRs. [file 12986_2020_510_MOESM1_ESM.docx]

**Ketogenic diet aggravates cardiac remodeling in adult spontaneously hypertensive rats**

**Table S1:** Ingredients of experimental diets

| Ingredient (g/kg diet) | Control diet | Ketogenic diet |
| --- | --- | --- |
| Casein | 100 | 180 |
| DL-methionine | 1.6 | 2.88 |
| Corn starch | 512.46 | 0 |
| Sucrose | 100 | 0 |
| Maltodextrin | 155 | 0 |
| Crisco | 25 | 440 |
| Cocoa butter | 0 | 150 |
| Corn oil | 25 | 85 |
| Cellulose | 35 | 9.19 |
| Calories per gram | 3.7 | 6.7 |

**Table S2:** Primers used in this study.

| Primers | Forward | Reverse |
| --- | --- | --- |
| IL-1β | TACCTATGTCTTGCCCGTGGAG | ATCATCCCACGAGTCACAGAGG |
| TNF-α | CTTCTGTCTACTGAACTTCGGGGT | TGGAACTGATGAGAGGGAGCC |
| IFN-γ | ATTTCCCTCCCCACTCCATTAG | CTGGTGACAGCTGGTGAATCA |
| GAPDH | CCTACCCCCAATGTATCCGTTGTG | GGAGGAATGGGAGTTGCTGTTGAA |


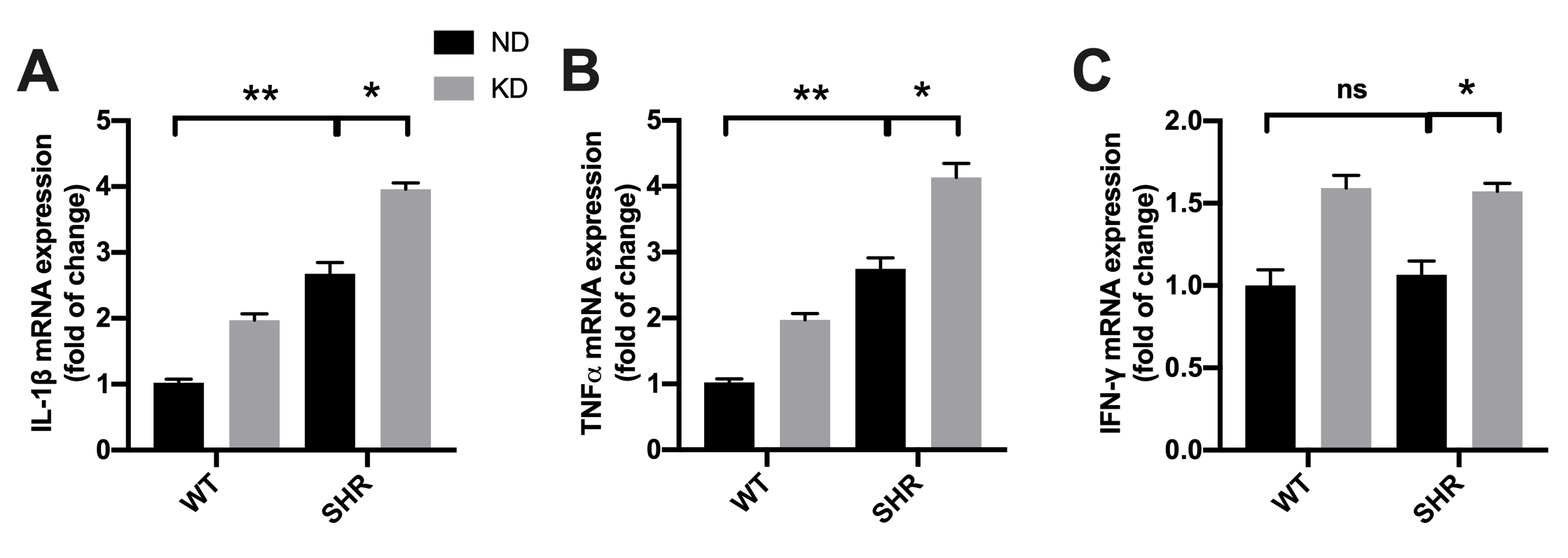


Figure S1. qRT-PCR was performed to measure the expression of IL-1β, TNF-α, and IFN-γ in the heart of WT and SHRs. Values are the mean ± SEM. n=4-6 for each group. *P < 0.05, **P < 0.01.
